# Is There a Relationship Between Mating and Pathogenesis in Two Human Fungal Pathogens, *Candida albicans* and *Candida glabrata?*

**DOI:** 10.1007/s40588-023-00192-8

**Published:** 2023-04-22

**Authors:** Tina Bedekovic, Jane Usher

**Affiliations:** MRC Centre for Medical Mycology, Department of Biosciences, Geoffrey Pope Building, University of Exeter, Stocker Road, Exeter, EX4 4QD UK

**Keywords:** *Candida albicans*, *Candida glabrata*, Mating, Evolution, Pathogenesis

## Abstract

**Purpose of Review:**

Human fungal pathogens are rapidly increasing in incidence and readily able to evade the host immune responses. Our ability to study the genetic behind this has been limited due to the apparent lack of a sexual cycle and forward genetic tools. In this review, we discuss the evolution of mating, meiosis, and pathogenesis and if these processes are advantageous to pathogens.

**Recent Findings:**

This review summarises what is currently known about the sexual cycles of two important human fungal pathogens, *Candida albicans* and *Candida glabrata.* This includes the identification of parasexual cycle in *C. albicans* and the observed low levels of recombination in *C. glabrata* populations.

**Summary:**

In this review, we present what is currently known about the mating types and mating/sexual cycles of two clinically important human fungal pathogens, *Candida albicans* and *Candida glabrata.* We discuss the evolution of meiosis using the knowledge that has been amassed from the decades of studying *Saccharomyces cerevisiae* and how this can be applied to fungal pathogens. We further discuss how the evolution of pathogenesis has played a role in influencing mating processes in human fungal pathogens and compare sexual cycles between *C. albicans* and *C. glabrata*, highlighting knowledge gaps and suggesting how these two fungi have evolved distinct mating niches to allow the development of disease in a human host.

## Introduction

The *Ascomycota* phylum is the most species rich of the Fungal Kingdom, covering a wide range of pathogens of both animals and plants [1]. Microbial pathogens have emerged not only independently in different phyla of the kingdom but also multiple times independently within the phylum [2•, 3•]. As more fungal genomes are sequenced and annotated, it is evident that there appear to be few, if any, truly asexual fungi [4, 5•, 6, 7•, 8, 9], with the machinery for both mating and meiosis appearing to be conserved [10, 11•, 12, 13]. Therefore, for pathogenic fungi, at least, a sexual nature is present and, in many cases, remains to be discovered under standard laboratory conditions. The rarity or cryptic nature of these sexual cycles leads to clonal populations with low levels of recombination events, which maybe be ancient or mitotic in nature [12], resulting in broad implications for the evolution of eukaryotic microbial pathogens. It should also be noted that solely clonal fungi are also rare, with molecular markers often revealing some degree of recombination having occurred [14]. Apart from footprints of recombination based on population genetics data, a further type of evidence indicative of a sexual cycle having occurred in most fungi comes from the apparent functionality of the mating type genes [15], a phenomenon seen in species without known sexual structures. In general, most ascomycetes exhibit mixed reproduction systems with signs of both sexual and asexual reproduction.

**Fig. 1:**
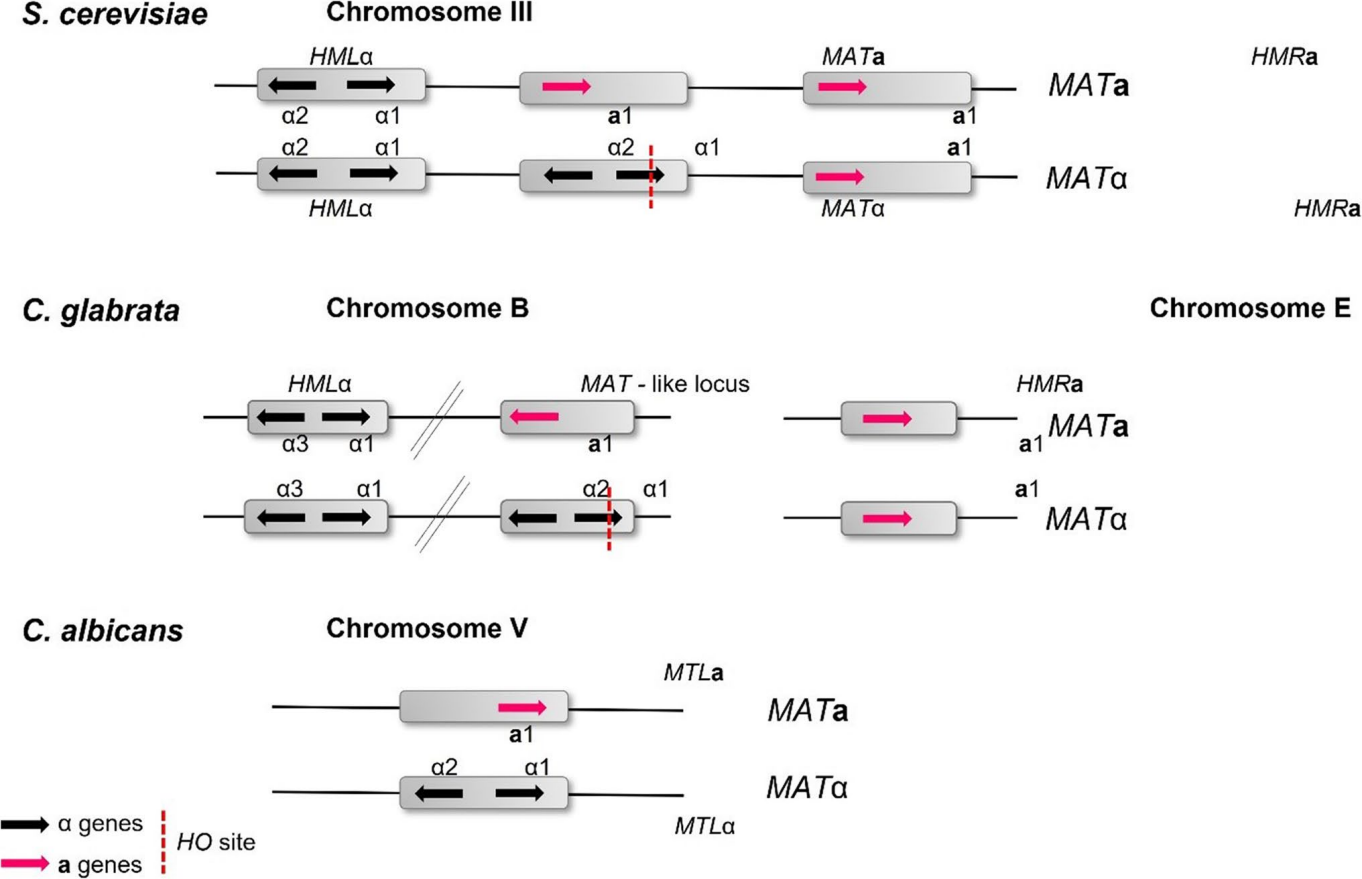
A schematic representation of mating-type loci in *S. cerevisiae*, *C. glabrata*, and *C.* *albicans*. In *S. cerevisiae*, mating-type loci are found on chromosome III and consist of *HML*α, *MAT*a/*MAT*α, and *HMR*a. Similarly, *C. glabrata* has 3 mating-type loci where *MHL*α and *MAT*-like a/α locus are found on chromosome B and *HMR*a on chromosome E. In contrast, *C.* *albicans* has only one *MTL*a/*MTL*α locus on chromosome V, without an *HO* endonuclease site. Reproduced with permission from Usher, The Mechanisms of Mating in Pathogenic Fungi—A Plastic Trait. *Genes* **2019**, *10*, 831, published by MDPI, 2019

At the molecular level, mating was first characterised and remains best studied in the yeast *Saccharomyces cerevisiae*; however, this is not a true representation for other fungi [16]. *S. cerevisiae* cells can be found to exist as one of three main mating types, **a,** α, and the diploid state **a/**α. Under favourable conditions, mating occurs between haploid **a** and α cell types, generating an **a/**α diploid [17]. Mating is activated by the presence of a pheromone, which binds to the Ste2 receptor in **a** cells or Ste3 receptor in α cells. For example, **a** cells mating pheromone “**a**-factor” indicates the **a** cell presence to neighbouring α cells. The α cells respond by growing a “shmoo” towards the source of the **a**-factor pheromone. The response of haploid cells to the mating pheromones of opposite mating types only facilitates mating between **a** and α cells but, in general, not between the cells of the same mating type. The phenotypic difference between **a** and α cells are due to specific sets of genes being transcribed and repressed in the different mating types [17–19]. These different sets of genes that characterise **a** and α cells are due to the presence of one of the two alleles on the *HML* or *HMR* and the *MAT* loci on chromosome III (Fig. 1), and the *HO* endonuclease on chromosome IV. The production of “shmoos” and mating in *S. cerevisiae* occurs via an all-or-none switch-like mechanism, therefore preventing the cells from unwise and energy inefficient mating. *MAT***a** haploids express the genes **a**1 and **a**2 from the *MAT***a** locus, with *MAT*α haploids expressing α1 and α2 from *MAT*α. Most yeast laboratory strains are heterothallic with stable mating types. However, some strains carry an active *HO* gene and are homothallic, indicating that as haploid cells they can switch mating type [16] via altering the genetic composition of the *MAT* locus to that of the opposite mating type. The progeny of the original cell can therefore mate and will form non-mating diploids with a silenced *HO* gene. In addition to the *MAT* locus, many strains also carry two complete but unexpressed copies of the mating-type genes at the silent loci *HML* and *HMR*, carrying alpha information and **a**-specific sequences, respectively. The mechanism of silencing *HML* and *HMR* loci is mediated by Sir2, a histone deacetylase, and its associated proteins. Mating-type switching process is stimulated by the *HO* endonuclease.

## Mating in *Candida albicans *and *Candida glabrata*

*C. albicans* is one of the best characterised fungi of the *Candida* (CTG) clade. Its pathogenic nature and close association with the human host have been studied for decades (Table 1) [20]. Its virulence is associated with the ability to grow in three different morphological forms: yeast, pseudohyphae, and hyphae [21–24]. Until recently, it was generally recognised as asexual in nature, as all known isolates were diploid and appeared to never have undergone meiosis. A *MTL* (mating type-like) locus that resembles the *MAT* locus of *S. cerevisiae* was identified by Hull, Raisner, and Johnson [25•], implying that mating may occur. It was also shown that genes known to play a role in mating and meiosis in *S. cerevisiae* are conserved in the *C. albicans* genome [26, 27].

**Table 1:** Genome comparison of *C. albicans* and *C. glabrata*

	*C. albicans*	*C. glabrata*
CTG clade	Yes	No
Ploidy	Diploid	Haploid
Number of ORFs	6203*	5271*
Number of Chromosomes	8	13
Mating types	**a**, α, **a**/α	**a**, α
Sexual cycle	Parasexual	Not observed naturally
Source	Human commensal	Human commensal/environment

^*^www.candidagenome.org

Mating in *C. albicans* was discovered in 2000 [25•]; since then, several studies have shown an elaborate mating system with many similarities, but many major differences also exists when compared to other fungi [5•, 24, 25•, 28–30]. The identification of an **a** and α version of the *MTL* locus revealed that the laboratory strain SC5314 is an **a**/α isolate with the size of the *MTL* locus of 9 kb compared to 0.7 kb in *S. cerevisiae*, attributed to three additional open reading frames at each locus [31]. These additional open reading frames were arranged in pairs, one member of which was present in *MTL***a** and one member in *MTL*α (Fig. 1, * www.candidagenome.org) [32].

*C. albicans* has been shown to mate in numerous reports using different approaches, but the collective theme among all studies, a normally heterozygous (**a**/α) strain of *C. albicans* (e.g. SC5314), was manipulated in the laboratory to create **a** and αstrains, which were then observed to mate. In the Johnson laboratory, **a**-type mating strains were constructed by deleting either the entire *MTL* locus or the *MTL*1 and *MTL*2 transcriptional regulators. Similarly, α-type mating strains were constructed either by deletion of the entire *MTL***a** locus or by deletion of the *MTL***a**1 and *MTL***a**2 genes [25•]. An alternative approach was used by the Magee laboratory [27], where they took advantage of the fact that the *MTL* of *C. albicans* resides on the chromosome V. Prior studies by Janbon et al. and Bennett et al. [29, 33] had made the discovery that one homolog of chromosome V was often lost during growth of *C. albicans* strains on certain selective media.

In *S. cerevisiae*, **a** and α cells are fully competent to mate and undergo sexual reproduction. This is not the situation in *C. albicans*, in which **a** and α cells must first undergo a phenotypic switch from the predominant white form to the scarcer and less stable opaque form to become mating competent. Miller and Johnson [34] subsequently described two key connections between white-opaque switching and mating. The ability of a *C. albicans* strain to undergo mating was regulated by the *MTL* locus and opaque phase, where **a** and α cells mated more efficiently than the same cells in the white phase. These findings illuminated why only a subset of clinical isolates of *C. albicans* underwent white-opaque switching; **a** and α cells, but not **a**/α cells, were competent for switching. It also explained the low level of mating previously reported, as white *C. albicans* cells were used in prior studies.

Although the parasexual cycle of *C. albicans* provides many of the advantages of a true sexual cycle, it is possible that *C. albicans* can also undergo meiosis. Examination of the genome of *C. albicans* has identified orthologs of several genes involved in meiosis in other fungi, including *DMC1* (*DLH1*), *SPO11*, and *HOP1* [5•, 35, 36]. Conversely, several important meiosis genes appear to be missing in the genome of *C. albicans*, suggesting that if meiosis occurs in *C. albicans* its structure and regulation is significantly different from that of other fungi [26, 37, 38].Aspects of mating and its regulation specific to *C. albicans* may have evolved to limit mating to specific locations in the mammalian body or to allow mating to take place under less than-optimal conditions [24, 29, 34, 39].

*C. glabrata* is now the second most commonly identified *Candida* species in a clinical setting and is phylogenetically closer to *S. cerevisiae* than to other *Candida* species [15, 40, 41•, 42–44]. It is part of the Nakaseomyces genus [45] and not the CTG clade that is home to *C. albicans* and many other *Candida* species [46]. This genus is currently made up of three yeasts isolated from the environment, *Nakaseomyces delphensis, Candida castellii*, and *Nakaseomycesbacillisporus*, along with two other pathogens, *Candida nivariensis* and *Candida bracarensis.* The members of this genus are both sexual and asexual in nature, with *C. glabrata* and *N. delphensis* containing the *HO* gene and *MAT*-like loci [41•, 47, 48].

The genomes of all six species have been sequenced and annotated and compared to *S. cerevisiae* [41•, 45]. As a general theme, all *Nakaseomyces* nuclear genomes are small, free from transposons, and contain fewer genes than *S. cerevisiae*. In contrast, their mitochondrial genomes, except for that of *C. glabrata*, are large and contain palindromic elements and GC inserts [41•, 47, 48, 49•, 50, 51]. The number of chromosomes ranges from eight in *C. castellii* to fifteen in *N. bacillisporus*. The “glabrata group”, composed of *C. glabrata*, *N. delphesis*, and *C. nivariensis*, has less variation in the number of chromosomes, with ten to thirteen chromosomes revealed by pulsed field gel electrophoresis [45]. In addition to the variable chromosome numbers, comparative analysis of the genomes revealed several gene losses and gains of species-specific genes. These *Nakaseomyces* specific events were used in comparative analysis to determine if these events were evolutionarily specific to *Nakaseomyces* group or specific to the “*glabrata* group” or indeed to *C. glabrata* itself [49•].

The *C. glabrata* genome has three *MTL* loci, and the *HO* gene has been previously reported in its genome, albeit the genome organisation is different from that of *S. cerevisiae,* with the *MAT* and *HML*-like cassette on chromosome B and the HMR-like cassette on chromosome E (Fig. 1) [7•, 52•, 53, 54]. In addition to isolates of both mating types being found, BG14 *MAT***a** genotype and CBS138 *MAT*α genotype are the most commonly utilised laboratory strains. Previous studies have set to examine the functionality of the genes involved in mating and meiosis in *C. glabrata* [7•, 42, 53, 55–58]. Gene expression profiling and functional complementation have revealed population structure evidence of recombination and orthologues of the majority of the genes involved in fungal sexual reproduction, including many of those missing in other *Candida* species. They found that the *MAT*α gene α1 was expressed in all *MAT*α strains and not in *MAT***a** strains, as expected [55]. However, the **a**1 gene was observed to be expressed in both *MAT***a** and *MAT*α strains; therefore, it was concluded that the *HMR***a** locus on chromosome V was not silenced in *C. glabrata*. One could therefore hypothesise that the genome re-organisation seen in *C. glabrata* is affecting gene expression, and this may play a role in the lack of a sexual cycle being observed. *C. glabrata* cells of both mating types express both **a-** and α- factor receptor genes, STE2 and STE3, respectively. However, upon the exposure of the cells to synthetic factor, no response was observed [13, 42]. When the *C. glabrata* pheromone was tested on *S. cerevisiae* cells, no effect was seen except when *sst2*∆ mutants were tested. *C. glabrata* cells are therefore able to produce pheromone that *S. cerevisiae* cells are sensitive to, but they themselves have an inhibited response.

## Evolution of Meiosis

Mating has yet to be obviously observed in *C. albicans* and *C. glabrata* in a laboratory setting; however, there is evidence of recombination at a genetic level in some clinical isolates [41•]. In *C. albicans*, mating between diploid isolates of opposite mating types produces a tetraploid, which can revert back to the diploid state via random chromosome loss in a parasexual cycle [5•]. Recombination occurs between the homologous chromosomes in a *SPO11*-dependent manner. The genes that have been identified in *S. cerevisiae* deemed essential for meiosis are present in both the genomes of *C. albicans* and *C. glabrata*. Therefore, there is no obvious reason as to why they cannot undergo meiosis, especially when recent work by Gabaldon et al. [52•] has shown evidence of recombination in the genome of *C. glabrata*.

Generally, it is assumed that genes will not be retained on the genome if the pathway is not functional. It can be postulated that these fungi do undergo recombination but only under very specific conditions, or that the gene function has been changed through evolution. The demonstration of recombination has been difficult to obtain; however, Gabaldon et al. [41•, 59•] have shown that through the sequencing of clinical isolates from around the world and constructing their evolutionary tree, seven subpopulations were observed. This demonstrated that clinical isolates of *C. glabrata* show more diversity than *C. albicans* and this diversity is not homogenous but organised in a set of diverse clades. This study confirmed the highly plastic nature of the *C. glabrata* genome with the ability to easily gain and loose genes, undergoing large regions of genomic reorganisation. They have also postulated that *C. glabrata* is probably environmental and sexual with the ability of favoring the exchange of genetic material to evade host immune responses.

Is it possible that the classical understanding of meiosis from *S. cerevisiae* creates bias in understanding these mechanisms in pathogenic fungi? Evidence from haploid *Candida* species is that sporulation is generally inefficient, resulting in fewer than four spores with aneuploid genomes [2•, 3•]. The parasexual cycle in *C. albicans* could be derived from a hybridisation event in an ancestral organism, and meiosis genes (such as *SPO11*) have evolved to drive recombination through other pathways more advantageous to the lifestyle of a pathogen.

## Evolution of Pathogenesis

Numerous studies, as previously mentioned, have identified expanded gene families in *Candida* species that are linked to pathogenesis, including carboxy-terminal glycosyl phosphatidylinositol (GPI)-anchored proteins mediating attachment of the fungal cell wall to host cells [21, 22, 60], the *ALS* gene family encoding cell wall proteins promoting adhesion to epithelial cells in *C. albicans* [27], and the EPA gene family in *C. glabrata* [61]. When comparing *C. albicans* and *C. glabrata* gene families related to pathogenesis, the genome of *C. glabrata* does not show the same expansion of genes. For example, *C. albicans* has 161 genes in 21 gene families annotated for pathogenesis, whereas *C. glabrata* contains only three genes from these groupings [49•].

This observation shows that *C. glabrata* has evolved alternative mechanisms from the majority of the CTG clade for seeding disease in the host environment. To further highlight the different routes to pathogenesis, *C. glabrata* does not utilise virulence traits such as hyphal growth and secreted proteinase activity that are hallmarks of pathogenesis in *C. albicans*. However, both do share some general features essential for virulence, such as phenotypic plasticity and varying numbers of host adhesins [11•, 23]. These two differently evolved routes to pathogenesis may also play a role in the cryptic sexual cycles observed. Is there an advantage to being a nonsexual haploid pathogen compared to a diploid pathogen that undergoes multiple morphological switches and a cryptic sexual cycle?

## Sex and Pathogenesis: Friends or Foes

In general, the concept of sexual reproduction is expensive and an inefficient use of energy and resources, and it reduces fitness through the maintenance of two different mating types. However, the sexual cycles have evolved, with most eukaryotes undergoing a full sexual cycle to allow for genetic diversity. The benefit of recombination and subsequent adaptations from a sexual cycle outweighs those of solely undergoing mitotic or clonal reproduction. Sexual reproduction does not appear to play a direct role in the infections caused by many human fungal pathogens; however, there does appear to be a difference in the pathogenicity between the two mating types in *C. glabrata* (Usher et al. in press, Microbiology Spectrum). In the diploid *C. albicans*, a small difference in virulence exists between strains that are heterozygous or homozygous at the mating type loci. However, loss of heterozygosity of non-*MTL* genes on chromosome 5 results in larger decrease in virulence and competitiveness during infection [62].

Population genetic studies show that *C. albicans* is primarily a clonal organism with low levels of recombination [63, 64]. However, strains heterozygous at the mating type locus can spontaneously generate mating-type homozygous strains either by loss of the mating type chromosome followed by duplication of the retained homologue or mitotic crossover. Therefore, the question remains: if heterozygous strains can spontaneously generate homozygous strains, why do they account for such a low percentage of the population? Indeed, why are there no reports of tetrads in clinical isolates of *C. albicans*? This may be due to the lack of meiosis genes in the genome, as previously discussed.

## Conclusions

Two of the main questions that are continually sought to be answered are why mating is not observed in many fungal species and how have these species evolved to retain homologs of the known genes essential for mating, sporulation, and meiosis. Is there a possible virulence role for the mating pathways beyond that of sex? For example, the formation of biofilms in *C. albicans* is often seen as a prelude to mating and the formation of drug-resistant biofilms. Have these genes been retained in the genomes of the *Nakaseomyces* as they are important for survival and evolving at a faster rate than non-sex genes? How have the *Nakaseomyces* adapted their sexual habits based on their differing environments?

These are the major questions that remain debatable as the research progresses in the field. It is essential that we try to determine the functionality of these genes in the fungal pathogens to determine if they have evolved a novel function as they may have a role in pathogenicity. The genomics age and the speed at which we can now perform genome sequencing and assembly of clinical isolates have a potential of revealing the complexity of mating loci of human fungal pathogens. Molecular population genomics has revealed that many of the human fungal pathogens undergo recombination, implying these “asexual” organisms may, in fact, be sexual and undergo unusual sexual cycles such as same-sex homothallic cycles or parasexual cycles, thus, providing enough or the “minimum” level of genetic diversity without affecting virulence potential.

Given that there are an estimated 150,000 fungal species described to date, only a small proportion cause disease in humans [65]. Is the pathogenicity in humans accidental on the part of the fungus? The virulence factors required for human disease, including mating type, could therefore also be required for environmental survival. We need to understand the reproduction and growth of human pathogenic fungi not only in the human host (one specific environment) but also in different environmental niches, for example the isolation of *C. glabrata* from avian samples [66]. Given the diversity of fungal taxa, we should not underestimate the potential of continued evolution and the emergence of new fungal pathogens.
